# Characteristics and influencing factors of early childhood teachers’ work stress and burnout: A comparative study between China, Ghana, and Pakistan

**DOI:** 10.3389/fpsyg.2023.1115866

**Published:** 2023-03-10

**Authors:** Siyuan Chen, Seth Yeboah Ntim, Yilun Zhao, Jinliang Qin

**Affiliations:** College of Child Development and Education, Zhejiang Normal University, Hangzhou, China

**Keywords:** stress, burnout, gender, comparative study, developing countries

## Abstract

**Introduction:**

Many studies have documented the stress and burnout experienced by early childhood teachers. However, few have focused on comparisons among countries, particularly developing ones. Meanwhile, female teachers, who are more sensitive and tend to provide emotional responses, are often overlooked as a major force of emotional involvement. This study examined the similarities and differences of early childhood teachers’ stress, burnout, and gender in China, Ghana, and Pakistan.

**Methods:**

This study adopted a cross-sectional design. The participants included 945 preschool and lower primary school teachers recruited from Zhejiang Province in China, the Ashanti Region in Ghana, and Punjab, Pakistan. The analyses were conducted using structural equation modeling. First, the study estimated all parameters separately and without constraints between the groups for all models. Second, the study compared the latent mean difference and of stressors and burnout between teachers’ personal and job characteristics. Third, a structural equation model was used to assess the relationship between teachers’ stressors and burnout.

**Results:**

Across the three countries, female teachers are more stressed out, with higher emotional demands and work-family conflicts, and are more prone to burnout with a greater level of emotional exhaustion and a lower level of personal accomplishments than their male counterparts are. Moreover, Chinese teachers were found to be the most stressed-out group with the highest level of burnout. In comparison to teachers in China and Pakistan, early childhood teachers in Ghana possess the lowest level of emotional demands. With the lowest level of emotional exhaustion and the highest level of personal accomplishments, Pakistani teachers were unlikely to experience burnout.

**Discussion:**

This study comparatively analyzed the characteristics of stress and burnout among ECTs in different cultural settings and educational systems in three developing countries (China, Ghana, and Pakistan), and revealed workplace characteristics and circumstances for ECTs. In addition, this study takes gender as the main influencing factor and explores its effect on ECTs’ stress and burnout, and it highlights and verifies "emotionality" in ECTs' profession. As a result, policymakers and stakeholders in different countries may be encouraged to improve ECE quality and the well-being of ECTs.

## Introduction

As a special type of teacher, early childhood teachers (ECTs) are not only responsible for supporting students’ academic work, but are also a major contributor to teacher-child interaction and emotional involvement (Jeon L. et al., 2019). The “emotionality” reflected in their care for children are the fundamental attribute of early childhood education (ECE) (Goldstein, 1997; Dalli, 2008; Jeon H. et al., 2019). In the past several decades, teaching has been considered one of the most stressful professions (Borg and Falzon, 1989; Kyriacou, 2001; Zhang et al., 2020), leading to a focus on teachers’ well-being (Wells, 2015; Kwon et al., 2020). Burnout is defined as cumulative emotional, mental, and physical exhaustion caused by work stressors that reduce one’s ability to maintain meaningful engagement at work (Schaufeli and Greenglass, 2001). According to Koch et al. (2015), nearly 56% of ECTs exhibit signs of burnout at work in Germany. However, not many studies have focused on ECTs’ stress and burnout. In recent years, their well-being has attracted much attention from society and simultaneously resulted in public reflections on the occupational stress of the teaching profession. Concerning ECTs’ stress and burnout, existing research primarily covers four aspects: (1) status and sources (Jeon H. et al., 2019; Lee and Wolf, 2019); (2) influencing factors such as job demands and resources, positive psychological capital (PsyCap), and emotional labor from a psychological perspective (Jeon H. et al., 2019; Schaack et al., 2020; Zhang et al., 2020); (3) the influence of individual traits and professional attributes, such as work ability, classroom climate, and emotional experiences (Lin et al., 2017; Jeon H. et al., 2019; Viotti et al., 2019); and (4) the relationship between occupational stress and burnout (Bottiani et al., 2019; Taylor et al., 2021). Stress can be considered either (1) a characteristic of the external environment acting on the individual; (2) the individual’s response or emotional state (psychological, physical, and behavioral) to the environment; or (3) an interaction between individuals and the environment (Cox, 1978; Kahn and Byosiere, 1992). According to Griffin and Clarke (2011), events in the environment that trigger the processes mentioned above are often referred to as stressors. A study from Romania analyzed ECTs’ stressors based on environmental and personal factors and found that major stressors included children’s problematic behaviors, high teacher-child ratios, excessive paperwork, issues unrelated to teaching, and poor communication with colleagues and parents (Clipa and Boghean, 2015). Similar outcomes were found by Lin et al. (2017) based on an analysis of ECTs’ emotional experiences at work: Teachers were more likely to have negative emotional experiences through a wide variety of daily work events, paperwork, and a negative interpersonal atmosphere. Although research has examined stressors, especially environmental factors, few investigations have compared stress characteristics in the context of different cultural backgrounds. In terms of ECTs’ stress, the focus on gender has been limited since most of the teachers are female. It remains to be seen how ECTs perform under stress in different cultures and how they experience differences because of gender.

Research supports the positive, direct relationship between stress and burnout (Zellars and Perrewé, 2001). To define burnout in terms of its causes, the influences of stressors are emphasized. According to Schaufeli and Greenglass (2001), with the accumulation of work stress, individuals stagnate and regress in their work; they subsequently experience emotional, mental, and physical exhaustion, which completes the state of burnout. Lin et al. (2017), in studying Chinese ECTs’ emotional experiences, found that high work intensity (such as long work hours and frequent extra work) and low emotional experiences in workplace arrangements (such as paperwork and a negative interpersonal atmosphere due to conflicts with colleagues and parents) lead to exhaustion and threats to teachers’ physical and psychological well-being. When teachers experience excessive work stress and burnout, they start to lose enthusiasm for their work and have negative emotional experiences that may further affect the quality of teaching and harm the immediate interests of the children in the class. Teachers with a high level of work stress and burnout are less likely to have higher professional commitment and provide children with positive emotional support (Buettner et al., 2016), positive teacher-child relationships (Koles et al., 2013; Ansari et al., 2022), and family-school relationships (Fantuzzo et al., 2012; Zhao et al., 2022).

According to traditional stress research, a comprehensive consideration of the relationship between the job decision-making dimension and job stressors has been lacking. Karasek (1979) developed the job demand-control model (JD-C), which effectively distinguishes two important individual factors—one’s job demand and control at work—and underscores the interaction effects between the two and their influences on professionals’ mental health. To enhance knowledge on job characteristics and personal well-being, the present study examines the separation of job demand as a stressor and job control as an outcome of special stress burnout in ECTs.

### Early childhood teachers’ work stressors

“Workload” has been widely applied in work and personnel psychology in recent decades. Gopher and Donchin (1986) defined mental workload as “the difference between the capacities of the information-processing system that are required for task performance to satisfy expectations and the capacity available at any given time” (pp. 41–43). More specifically, Jex (1988, p. 11) defined it as “the operator’s evaluation of the attention load margin (between motivated capacity and the current task demands) while achieving adequate task performance.” Applying this conceptual framework to measurement and job demand management will contribute to a clearer understanding of the different effects of employees’ capacity and motivation constraints on their job performance and related emotional states (Macdonald, 2003). It is concluded that the level of workload is not only determined by the amount of time and effort spent on certain tasks and the amount of resources allocated between tasks, but also by the level of motivation an individual has (Charles and Nixon, 2019; Dehais et al., 2020). Several studies have confirmed that ECTs have a heavy workload, mainly in terms of time pressure and work-related arrangements. A study from China found that ECTs work 9.28 h and an extra 1.46 h per day on average (Lin et al., 2017), while in the US, ECTs are required to work more than 8 h per day in the classroom on average (Diana et al., 2020). The research highlights ECTs’ very limited capacity within their unique role, as well as their emotional involvement, which may affect their psychological and emotional capacity. Multifaceted affairs and paperwork are major challenges. Lin et al. (2017) indicated that ECTs have 16 different tasks to complete both during and after work, including a great deal of paperwork that may evoke negative emotions. Likewise, Sakai et al. (2014) found that ECTs experienced an expansion of their job roles and responsibilities. The aforementioned studies suggest a clear path that limits teachers’ motivation at work.

Emotional demands at work entail consistent emotional involvement and efforts required by one’s job (Zapf, 2002). As for meeting organizational goals, Hochschild (1983) introduced the concept of “emotional labor” which refers to “the management of feeling to create a publicly observable facial and bodily display” (p. 7). Hochschild differentiated between physical and emotional labor based on employees’ intentions regarding the use of physical motions and managing one’s feelings. Grandey (2000) further explored the framework of emotional labor and developed an interactive model where individuals regulate emotions at work for emotional management. The mechanism consists of four components: (1) situational cues, including interactional expectations and emotional events that elicit individuals’ emotional labor; (2) the emotional regulation process; (3) influencing factors such as organizational well-being and personal well-being (e.g., burnout); and (4) the consequences related to both. As situational cues that contribute to emotional labor, interactions with students, parents, and colleagues occupy teachers’ daily work. The intensity, frequency, duration, and variety of experienced emotions determine one’s emotional performance, which is a core work requirement (Brotheridge and Lee, 2002). A study from China indicated that ECTs are required to express highly intensive, long-lasting, and diverse emotions during their daily tasks as educators (Zhang et al., 2020). As a result, different types of job requirements and their corresponding self-regulation processes are subject to competition for psychological resources when these resources are limited (Hülsheger and Schewe, 2011; Charles and Nixon, 2019). For instance, in terms of a negative emotional event, studies highlighted the positive relationship between children’s problematic behaviors in the classroom and teachers’ emotional stress levels (Friedman-Krauss et al., 2014; Jeon H. et al., 2019). Teachers may suffer from emotional exhaustion as a result of coping with children’s problematic behavior. Alternatively, if a teacher fails to regulate their emotions and to provide emotional support to children at work, they may experience stress (Buettner et al., 2016).

Considerable research has focused on work–family conflict, and various process models have been proposed to better understand the bi-directional nature of conflict (Greenhaus and Parasuraman, 1986; Adams et al., 1996; Kinnunen and Mauno, 1998). The conceptual model indicates that in their reciprocal relationship, work and family interfere with each other when the roles and obligations of both parts are unfulfilled yet interconnected (Adams et al., 1996; Parasuraman et al., 1996; Frone et al., 1997). Certain antecedents are widely recognized as factors that lead to a hostile relationship, such as role ambiguity, role conflict, time demands, and role involvement on both sides (Nikmah et al., 2020; Gu and Wang, 2021). A direct and positive relationship has been found in role conflict, role ambiguity, time demands, and work–family conflict, while high role involvement on both sides predicts a stressful relationship (Nikmah et al., 2020; Gu and Wang, 2021). Consequently, the conflicted relationship will ultimately produce dissatisfaction in both work and family contexts, as well as personal well-being (Bedeian et al., 1988). As a profession with a high level of emotional involvement and demands, ECTs are at great risk of work–family conflicts (Bulger et al., 2007; Gu and Wang, 2021). Teachers who are deeply involved in interactions at work should take time to rest or seek support from family after work in order to regulate their emotions. However, this is not always the case. Lin et al. (2017) found that ECTs continue to have negative emotional experiences after the end of their workday. Teachers might use extra time at home to complete tasks such as paperwork, leaving them unable to seek emotional support from family members who do not understand their work role (Li, 2015), or experience conflict with family roles since teachers also have family obligations as members of a female-dominated profession (Noor and Zainuddin, 2011; Li, 2015; Gu et al., 2020). Family obligations may drain the resources of ECTs, sometimes even taking up part of their work time to deal with family matters, causing interruptions at work. Thus, it is sometimes difficult for teachers to maintain a positive emotional demeanor during interactions at work.

### Early childhood teachers’ stress and burnout

Various factors affect job burnout, with researchers positing that the most direct cause of job burnout is workplace stress (Zellars and Perrewé, 2001). Some scholars even consider a causal relationship between stress and burnout: Burnout is the most direct outcome of workplace stress (Melamed et al., 2011). Regarding definitions, researchers tend to focus on a direct relationship. For instance, Maslach (1986) defined burnout as a response to stress comprised of three dimensions: (1) emotional exhaustion; (2) depersonalization; and (3) reduced personal accomplishments; this definition is still widely used today. Blase (1982) asserted that job burnout refers to emotional, attitudinal, and physical exhaustion caused by workplace stress. Likewise, Mearns and Cain (2003) found that stress predicted burnout.

While it is not always the case, the relationship between work stress and burnout could be far more complex. Studies found that moderate work stress is conducive to maintaining teachers’ enthusiasm for their work, and burnout will occur only when their stress is either above or below a certain level (Gmelch, 1983; Freire et al., 2020). Similarly, it is pointed out that work stress, as an influencing factor, does not directly lead to burnout (Pines and Keinan, 2005; Freire et al., 2020).

Based on the above studies, moderate work stress contributes to teachers’ motivation and work efficiency, while long-term accumulated stress may in turn lead to adverse individual physical and psychological reactions as well as burnout.

ECTs are exposed to stressors that lead to burnout and decrease their well-being (Zellars and Perrewé, 2001; Hong and Zhang, 2019). According to Maslach and Leiter (2008), emotional exhaustion, especially among ECTs, involves a lack of energy and a sense of emotional resource depletion, as well as persistent physical, psychological, or emotional efforts that cause stress when resources are exhausted. As Jennings and Greenberg (2009) pointed out, teachers can play a critical role in creating a positive social–emotional environment for students and can thus contribute to their social–emotional development as well. When teachers are emotionally exhausted, it is difficult for them to respond positively to children’s emotional needs; they are more likely to have conflicts with children, especially when children display problematic behaviors (Whitaker et al., 2015). Teachers’ emotional exhaustion is negatively related to effective communication with colleagues and parents (Zhang et al., 2020) as well as student engagement in the classroom (Leiter and Maslach, 1999; Jeon H. et al., 2019). These findings suggest that there is a high level of emotional demand in the profession, and teachers are at great risk of emotional exhaustion. Furthermore, emotional exhaustion is the most critical component of measuring burnout because it is the first step to developing burnout, leading to higher levels of depersonalization and fewer achievements (Byrne, 1994; Maslach et al., 2001). It has been demonstrated, for example, that emotional exhaustion negatively affects a teacher’s motivation to teach (Hakanen et al., 2006; Pishghadam et al., 2019), preparation and engagement in the classroom (Leiter and Maslach, 1999; Jeon H. et al., 2019), self-efficacy (Schwarzer and Hallum, 2008; Kremanshahi and Pishghadam, 2022), and commitment to the profession (Buettner et al., 2016).

### Comparisons between China, Ghana, and Pakistan

There has always been a strong interest in comparative studies in educational research; however, most studies focus on comparisons between developed and developing nations or between developing and developed countries. Very few studies have been conducted exclusively on developing nations, especially those focusing on teachers. In developing countries, some common characteristics influence the establishment and development of educational systems, such as having a dualistic economy, being dominated by agriculture (Todaro, 1981), and having a single industrial structure (Wickrama and Mulford, 1996) to be reformed and reflected by politics. Zong et al. (2022) found that the economic growth theory, which centered on the goals and models of Western powers, does not apply to the reality of developing nations. The development process should be based on the needs of humans and society, including cultural and spiritual ones. To facilitate these needs, education plays a vital role, especially ECE as a foundation. Expanding abilities to interact and form relationships with preschool children contribute to school readiness and future success (Domitrovich et al., 2007; Denham et al., 2012). In recent years, developing states have become committed to making ECE more accessible and the scale has been growing, especially since China is one of the fastest developing nations for ECE in Asia, as is Pakistan (Huo and Sun, 2015). In China, Confucianism, with benevolence at its core, has a profound influence on teachers, instilling the values of tolerance, courtesy, and sacrifice in the way that ECTs treat children and others, thus generating emotional demands for teachers at work. As a national religion, Islam emphasizes peace, obedience, and being active (Du, 2012). Under such circumstances, work status and ECTs’ stress in both China and Pakistan are worth exploring. In contrast, as a post-colonial African state, Ghana’s education reform has always attached great importance to the input of basic education, including ECE, since gaining independence in 1957. In 2002, kindergarten was included in the formal Free and Compulsory Universal Basic Education (FCUBE) system to fulfill the government’s commitment to reduce the financial burden on poor families accessing early childhood services (Agbenyega, 2018). Interestingly, most Ghanaians are Christian and because of Ghana’s colonial history, religious education has always been a key component of its educational system. Independence (individualism) is also rooted in Christian education (Addai-Mununkum, 2014). In terms of social values, Hofstede carried out a large cross-cultural survey among 78 countries to explore social values, and found that compared to individualism, Ghana was a relatively collective country with a rank of 58 on individualism (Hofstede et al., 2005). This suggests that Ghanaians are integrated into groups and have respect for collective values as group members, while favoring individual independence (Hofstede, 2011). This study takes the educational comparison among developing countries as the basic background, focuses on the work characteristics and well-being of ECT, and selects China, Pakistan and Ghana as the research objects. The main reasons are as follows: First, China, Pakistan and Ghana obtain long-term friendly and strategic cooperation relationships. Educational exchanges can maintain traditional friendship and deepen humanistic exchanges (Peng and Zhang, 2021). Second, educational exchanges in the three countries are conducive to maintaining regional peace and stability and realizing regional prosperity (Peng and Zhang, 2021). Third, in the process of educational exchange, culture is always an indispensable and important factor. As one of the sources of a country’s soft power, culture plays an increasingly prominent strategic role in national exchanges (Xiang, 2011). The three countries studied are all developing nations with collective cultures and are experiencing the rapid expansion of ECE. As such, ECTs’ stress and burnout in different cultural contexts are worth exploring. Differences and similarities may provide references for teachers’ development and well-being in all three nations. Research in all three countries has examined the relationship between teachers’ stress, burnout, and demographics, but no studies have compared these three states to each other to investigate the relevant relationships and characteristics.

### The present study

Preliminary studies indicate that ECTs are stressed when coping with demands such as long work hours, a wide range of daily tasks (including paperwork), and high emotional involvement with children and relative adults alike; they are particularly vulnerable to burnout. ECTs’ work stress and burnout should be investigated in terms of their characteristics and factors that contribute to their burnout. Most studies have explored the circumstances of ECTs’ work stress and found differences in dimensions based on both personal and job-related traits such as age, education level, work experience, and teaching level (Hakanen et al., 2006; Sakai et al., 2014; Lin et al., 2017). Few studies have compared ECTs’ situations across different nations, especially developing ones. The purpose of this study is to examine the characteristics and influencing factors of ECTs’ work stress and burnout in China, Ghana, and Pakistan to address the following questions:

1. What are the common characteristics of job demand stressors and burnout among ECTs in different educational systems (China, Ghana, and Pakistan)?
2. What are the major differences among ECTs in terms of work stress and burnout in different educational systems (China, Ghana, and Pakistan)?
3. Why might there be similarities and differences among ECTs regarding work stress and burnout in different educational systems (China, Ghana, and Pakistan)?

## Method

### Instrument

#### Teacher stress

Teachers’ stress in the ECE work environment was measured with three constructs (workload, emotional demand, and work–family conflict) adapted from and validated by Aboagye et al. (2018), who performed cross-cultural studies. The concept of workload was originally developed by Siegrist et al. (2004) and consists of 5 items (e.g., “My work requires too much from me”). Participants were asked to rate their answers on a 4-point scale according to the frequency of their work (1 = never and 4 = frequently). Emotional demand was measured based on 7 items (e.g., “My work demands a lot from me emotionally”) developed by Veldhoven and Meijman (1994). Teachers were asked to rate the frequency of events occurring on a 4-point scale (1 = never to 4 = always). Lastly, work–family conflict was measured using 5 items developed by Frone et al. (1996). The items included statements such as “My work takes up time that I’d like to spend with my family,” and they are measured on a 4-point scale ranging from 1 = never to 4 = very often. The Cronbach’s alpha values for the sub-scales of occupational stress (i.e., emotional demand, workload, and work family conflict in order) were 0.90, 0.79, and 0.87 respectively, for China; 0.84, 0.84, and 0.81 respectively, for Ghana; and 0.91, 0.82, and.77 respectively, for Pakistan. These sub-scales of occupational stress are reliable and valid in cross-cultural research (see Aboagye et al., 2018).

#### Teachers’ burnout

Teacher burnout was assessed using 22 items on a Likert scale developed by Maslach and Jackson (1981) and adapted by Aboagye et al. (2018) in China, Ghana, and Pakistan. The Maslach Burnout Inventory–Educator’s Survey (MBI–ES) consists of three sub-scales: (1) emotional exhaustion; (2) depersonalization; and (3) personal accomplishments. Emotional exhaustion consists of 9 items (e.g., “I feel emotionally drained from my work”), depersonalization consists of 5 items (e.g., “I feel I treat some students as if they were impersonal objects”), and accomplishments contain 8 items (e.g., “I have accomplished many worthwhile things in this job”). Responses were given on a 7-point Likert scale ranging from 1 = never to 7 = every day. Cronbach’s alpha values for the three sub-scales of teacher burnout (i.e., emotional exhaustion, depersonalization, and personal accomplishment) were 0.82, 79, and 0.76 respectively, for China; 0.81, 0.79, and 0.78 respectively, for Ghana; and 0.81, 0.76, and 0.73 respectively, for Pakistan. Reliability and validity of the MBI–ES have been established in previous studies (Boles et al., 2000; Aluja et al., 2005; Schwarzer and Hallum, 2008; Qiao and Schaufeli, 2011; Aboagye et al., 2018).

### Participants

The participants consisted of 945 teachers (332 male and 613 female): 350 (37.04%) Chinese, 285 (30.16%) Ghanaians, and 310 (32.16%) Pakistanis. These preschool and lower primary teachers were recruited from both public and private schools across cities in China’s Zhejiang Province, Ghana’s Ashanti Region, and Punjab in Pakistan. These provinces, regions, and states host various ethnic, cultural, technological, economic, and administrative activities of the region, with schools enlisting pupils and teachers from different economic and socio-cultural backgrounds. Recruitment took place in March 2017. The printed questionnaire was then given to the participants to answer voluntarily within 20 min; souvenirs were given upon completion of the questionnaire.

The demographic and job characteristics were designed to establish the variables, including gender, age, education level, work experience, school type, class size, and teaching level. The respective genders of the teachers were coded as 1 for males and 2 for females. Age was coded as 1 for 30 years or younger, 2 for 30 to 39 years, and 3 for 40 years and above. Education level was categorized into four levels: junior high school and below; senior high and technical schools; bachelor’s degree/college; and master’s degree and above. Teachers’ job characteristics, such as work experience, were categorized into three levels: 1 = 6 years or less; 2 = 7 to 15 years; and 3 = 15 years or more. School type was classified as 1 = public school and 2 = private school. Class size was categorized as: 15 students or less; 16 to 25 students; 26 to 35 students; and 35 or more students. Teaching level was categorized as: nursery; kindergarten; and grade one and above. Females constituted the majority of the participants (613 women, or 64.9% of the sample). Most of the teachers were younger than 30, followed by those aged 30 to 39, then 40 and older. Most of the teachers held a bachelor’s degree or above (602 individuals, or 63.7% of the sample). Most of the teachers taught 26 or more students in a given classrooms (759 individuals, or 82.2% of the sample; Table 1).

**Table 1:** Descriptive analysis of personal and job-related characteristics.

	Country	Sex	Age	Education level	Job experience	School type	Class size	Teaching level
	China Pakistan Ghana	Male Female	<30 years 30 to 39 ≥40	Junior high School High school/technical school College/undergraduate Master’s +	6 years or less 7 to 15 >15 years	Public Private	≤15 16 to 25 26 to 35 >35	Nursery Pre-school First grade and above
*N* (%)	350 (37.04%) 310 (32.80%) 285 (30.16%)	332 (35.1%) 613 (64.9%)	385 (40.7%) 333 (35.2%) 227 (24.1%)	172 (18.2%) 171 (18.1%) 310 (32.8%) 292 (30.9%)	474 (50.2%) 277 (29.3%) 194 (20.5%)	586 (62.0%) 359 (38.0%)	61 (6.5%) 107 (11.3%) 383 (40.5%) 394 (41.7%)	186 (19.7%) 566 (59.8%) 193 (20.5%)
Mean			32.89		8.93			
SD			8.16		7.24			

### Analytical plan and results

The analyses were conducted in three parts using structural equation modeling (SEM) (Amos). The first step involved multi-group confirmatory factor analyses (MGCFAs) on data to test the measurement invariance (MI) of the stressor factor model and the burnout factor model. Two invariance types were examined: (1) configural and (2) metric (Meredith and Teresi, 2006; Chen, 2007). This study used an invariance test to assess the equivalence of the measurement model for different groups (i.e., country, gender) following the procedure outlined by Bollen (1989).

First, the study estimated all parameters separately and without constraints between the two groups (i.e., configural invariance) for all models. The unconstrained model showed acceptable fit across groups. Further, the second model (metric invariance) was scrutinized, and the entire factor loadings were constrained equally between groups and estimated simultaneously. A chi-square difference test was employed to compare the models. Based on Chen (2007) and Cheung and Rensvold (2002), the ΔCFI, ΔRMSEA, and ΔSRMR indices were investigated for MI model fit. Given the sample size (*N >* 500) approximately distributed across groups in testing metric invariance, a change of ≤0.010 in CFI supplemented by changes of ≤0.015 in RMSEA and ≤ 0.030 in SRMR indicated equivalence or invariance (see Table 2). For the second step, we compared the latent mean difference of the continuous variables (stressors and burnout) between teachers’ personal and job characteristics (see Table 3). Third, a structural equation model was used to assess the relationship between teachers’ stressors and burnout (see Table 4).

**Table 2:** Multi-group configural and metric invariance of the stress scale and burnout.

Model	*χ* ^2^	D*f*	CFI	TLI	RMSEA	RMR	Δ*χ*^2^	Δd*f*	*p*-Value	ΔCFI	ΔRMSEA	ΔRMR
Country												
Stress												
Configural	686.830	308	0.950	0.934	0.036	0.056						
Metric	1283.409	340	0.875	0.850	0.045	0.054	596.579	32	0.000	−0.075	0.009	−0.002
Burnout												
Configural	232.537	108	0.954	0.929	0.035	0.047						
Metric	281.176	130	0.944	0.929	0.035	0.052	48.639	22	0.001	−0.01	0.00	0.005
Gender												
Stress												
Configural	509.273	214	0.931	0.912	0.038	0.058						
Metric	609.951	231	0.911	0.895	0.042	0.077	100.678	17	0.000	−0.02	0.004	0.019
Burnout												
Configural	229.799	75	0.941	0.914	0.047	0.054						
Metric	214.618	83	0.950	0.934	0.041	0.059	15.181	8	0.056	0.009	−0.006	0.004

**Table 3:** Latent factor mean comparisons across country and gender for stress and burnout.

	Stress			Burnout		
	Workload	Emotional demands	Work–family conflict	Emotional exhaustion	Depersonalization	Personal accomplishments
Country						
China ^a^						
Ghana	−0.11	−2.10***	−0.18*	−0.13	−0.06	0.25*
Pakistan	−0.45***	−0.25	0.03**	−0.69***	0.28**	1.15***
Gender						
Male ^a^						
Female	0.05	0.21***	0.11*	0.26***	−0.35***	−0.19**

*p < 0.05, **p < 0.01, ***p < 0.001.

**Table 4:** The direct effects of stressors on burnout.

Unstandardized estimates (SE)									
	Ghana			China			Pakistan		
	EE	DP	PA	EE	DP	PA	EE	DP	PA
Sex	0.31† (0.16)	0.06 (0.11)	−0.21** (0.08)	0.15 (0.17)	−0.18 (0.16)	−0.03 (0.11)	0.08 (0.28)	−0.52† (0.27)	0.32* (0.13)
Age	0.01 (0.01)	−0.01 (0.01)	−0.00(0.00)	0.02 (0.02)	−0.01 (0.01)	−0.00 (0.01)	−0.05** (0.02)	−0.00 (0.02)	−0.01† (0.01)
Edu	−0.02 (0.08)	−0.04 (0.06)	0.05 (0.04)	−0.11† (0.06)	−0.05 (0.06)	0.03 (0.04)	−0.14* (0.06)	−0.02 (0.06)	0.02 (0.03)
WkExp	−0.01 (0.01)	−0.00 (0.01)	0.00 (0.00)	−0.06*** (0.02)	−0.01 (0.02)	0.01 (0.01)	0.04* (0.02)	0.01 (0.02)	0.01 (0.01)
School type	0.14 (0.16)	0.08 (0.11)	−0.04(0.08)	0.07 (0.15)	−0.21 (0.14)	0.15 (0.09)	−0.10 (0.29)	−0.17 (0.28)	−0.11 (0.14)
Class size	−0.15† (0.10)	−0.08(0.05)	0.06(0.04)	−0.11(0.07)	−0.09(0.07)	−0.00(0.05)	−0.18*(0.08)	−0.01(0.07)	−0.00(0.04)
EDM	−0.10(0.15)	−0.03(0.10)	0.08(0.07)	0.32*(0.14)	0.23†(0.13)	0.07(0.09)	0.26†(0.14)	0.14(0.13)	0.06(0.06)
WFC	0.10(0.10)	−0.05 (0.06)	−0.00 (0.04)	0.61*** (0.08)	0.23** (0.08)	0.08 (0.05)	0.53*** (0.08)	0.37*** (0.07)	−0.06† (0.04)
WKL	0.11 (0.13)	0.02 (0.09)	0.12† (0.06)	0.45*** (0.13)	0.10 (0.12)	0.16* (0.08)	−0.04 (0.11)	0.03 (0.10)	0.04 (0.05)
*R* ^2^	0.05	0.02	0.08	0.39	0.12	0.07	0.24	0.22	0.10

EDM, Emotional demands; WFC, Work–family conflict; WKL, Workload; EE, Emotional exhaustion; DP, Depersonalization; PA, Personal accomplishment; WkExp, Work experience; Edu, Education level. †*p* < 0.10, *p < 0.05, **p < 0.01, ***p < 0.001.

### Structural invariance tests across country and gender for the stress and burnout models

The measurement models of the stressor and burnout scales satisfied acceptable fit, mostly at the configural level. This is evident from the CFI, TLI ≥ 0.90, and SRMR and RMSEA ≤0.08 for the model. The acceptable configural model fit can be interpreted as evidence of configural invariance of the stress and burnout scales across the three participating countries, as well as across genders; one’s citizenship and gender are essential criteria for determining the optimization method (Asparouhov and Muthén, 2014). That is, when there were no equality constraints on the factor loadings, neither the intercepts of the stress or burnout models demonstrated an acceptable measure of the latent factor across country and gender. However, the stress and burnout models did not meet the metric invariance requirement due to excellent fit. This is because the results of Δ*χ*^2^ revealed some degree of measurement non-invariance across country and gender for both the stress and burnout models upon comparing the configural and metric models with Δ*χ*^2^. Additionally, ΔCFI, ΔTLI and ΔRMSEA were not within the recommended standards of measurement invariance proposed by Chen (2007). For these, both the stress and burnout models were concluded to be variant across country and gender (Chen, 2007).

### Results of the latent mean differences of stress and burnout

Using China as the reference group, the analyses revealed a statistically significant difference between China and Ghana, with Ghana scoring a lower mean on stressors (i.e., emotional demands and work–family conflict), but scoring a higher mean than China on personal accomplishments. Further, Pakistan and China had a significant difference for all the three components of burnout, with Pakistan scoring higher on depersonalization and personal accomplishments, but lower on emotional exhaustion than China.

Males were used as the reference group. The results indicated a statistically significant difference between male and female teachers for work stressors, with females scoring a higher mean on emotional demands and work–family conflict than their male counterparts do. Further, a statistically significant difference was found in all three sub-scales of burnout between male and female teachers, with female teachers scoring significantly higher on emotional exhaustion, but lower on depersonalization and personal accomplishments compared to their male counterparts.

SEM was employed to test the relationships between teachers’ stressors (workload, emotional demands, and work–family conflict) and (1) emotional exhaustion; (2) depersonalization; and (3) personal accomplishments. The model provided acceptable fit indices to the data: *χ*^2^ = 1890.21, df = 1,179, *χ*^2^/df = 1.60, RMSEA = 0.03, SRMR = 0.04, and CFI/TLI = 0.93/0.92. Emotional demands had a significant and positive effect on emotional exhaustion (*b* = 0.32, SE = 0.14, *p* < 0.05) for the Chinese portion of the sample. Further, work–family conflict had a significant and positive effect on emotional exhaustion (China: *b* = 0.61, SE = 0.08, *p* < 0.001; Pakistan: *b* = 0.53, SE = 0.08, *p* < 0.001), and depersonalization (China: *b* = 0.23, SE = 0.08, *p* < 0.01; Pakistan: *b* = 0.37, SE = 0.07, *p* < 0.001). Last, workload had a significant and positive effect on emotional exhaustion (*b* = 0.45, SE = 0.13, *p* < 0.001) for the Chinese portion of the sample, and personal accomplishments (Ghana: *b* = 0.12, SE = 0.06, *p* < 0.10; China: *b* = 0.16, SE = 0.08, *p* < 0.05). The model explained 5%, 2%, and 8% of the variance for Ghana; 39%, 12%, and 7% of the variance for China; and 24%, 22%, and 10% of the variance for Pakistan regarding the variability of emotional exhaustion, depersonalization, and personal accomplishments, respectively (see Table 4).

## Discussion

ECTs are the main participants in early education, where teacher-child interactions not only promote the healthy growth of children, but also guarantee the sustainable development of teachers. Peisner-Feinberg et al. (2001) found that process quality stimulates child development; this involves sensitivity, responsiveness, positive interactions, emotional support, and instructional support provided by teachers. Work stress and emotional exhaustion are negatively related to a teacher’s ability to provide emotional support and positive child behavior management (Hamre, 2014). In this case, ECTs’ stress and burnout are not only the key factors of ECE quality but also the main reasons affecting teachers’ stability and professional growth. Although previous studies have paid more attention to the impact of job demands on ECTs, few have explored the reasons behind them. At the same time, the comparison of teachers’ work characteristics and stress in different cultural and educational contexts is rarely discussed. Based on cultural perspectives, this study compared the similarities and differences of stress and burnout among ECTs in three developing countries (China, Ghana, and Pakistan) and focused on analyzing the characteristics and influences of teacher stress and burnout. The main conclusions are as follows.

For similarities, although ECTs in the three cultural contexts have their own characteristics, the structure and influencing factors of burnout are consistent. With the factorial validity of MBI–ES being verified across China, Ghana, and Pakistan (Aboagye et al., 2018), it sets the foundation for cross-cultural research on teachers’ burnout and well-being, and leaves spaces for further comparative exploration.

Compared with male teachers, female ECTs in the three countries exhibited a higher level of work stress (especially emotional demands and work–family conflict) and emotional exhaustion, as well as a lower level of depersonalization. From the perspective of gender differences and job roles, there may numerous reasons. First, for coping strategies, compared to female teachers, male ECTs engage in physical activity more often, as well as more diverse and intense forms of exercise in both work and life. Physical exercise plays a positive role in coping with individual psychological stress and boosts one’s tolerance for frustration (Li, 2013). Second, female ECTs are more emotionally involved in work. From a sociological angle, women are seen as more emotional than men are, with this belief applied across cultures (Heesacker et al., 1999; Hess et al., 2000), including with regard to the intensity of emotional experiences (Davis et al., 2012; Ding et al., 2021). Similar results have been partially confirmed from a physiological standpoint, indicating that women are more emotionally reactive than men in terms of psycho-physiological reactions (Bradley et al., 2001; Chen et al., 2018). However, some studies have drawn different conclusions, finding no significant differences between men and women in their psychological and physiological responses (Katkin and Hoffman, 1976; Vrana and Rollock, 2002). Although outcomes are mixed, the emotional regulation perspective may provide a new way of analyzing problems. For example, compared with women, men show less of an increase in prefrontal activity and more of a decrease in the amygdala when reappraisal is applied to reduce negative emotions (McRae et al., 2008; Jentsch et al., 2019). This suggests that men put less effort into emotional regulation due to the use of automatic emotional regulation, and thus experience increased neural efficiency. Cognitive reappraisal is an efficient method to cope with stress and emotional regulation, while stress (particularly the stress hormone cortisol) facilitates emotional regulation processes (Kinner et al., 2014; Jentsch et al., 2019). Moreover, increases in stress-induced cortisol mitigate the rise in negative effects normally observed following stress (Het and Wolf, 2007; Het et al., 2012). Further, compared with female teacher participants, stress has been found to decrease male participants’ emotional arousal and increase the likelihood of success in reappraisal, which contributes to the process of emotional regulation, whereas no significant influences of stress were found for the reappraisal success rate in female participants. Similar correlations were observed between cortisol secretion and reappraisal level, but only in male participants (Katja et al., 2020). From the perspective of neuroscience, when coping with stress, men tend to be more rational than women do and to provide non-emotional cognitive appraisals in stressful situations, thus reducing the cost and difficulty of emotional regulation. Interestingly, cortisol secreted by the HPA axis (when a person is stressed) improves the success rate of emotional regulation in men, which establishes a physiological advantage when coping with emotions, especially negative ones. Hence, in general, women have a higher risk of emotional involvement and exhaustion when their jobs are emotionally demanding. This is especially the case for ECTs.

In terms of job roles, as a minority among ECTs, male teachers have a positive impact on children’s overall development. Existing studies mainly focus on the promotion of male teachers in the development of children’s motor skills and the formation of gender consciousness, which is closely related to educational practices. Gender differences exist in both work arrangements and self-choice in early childhood programs (Sumsion, 2005). Further, masculinity involved in child play is performed by male teachers for gross motor skills, such as climbing, jumping, and running, activities that are popular among children (Wang and Chai, 2008). Female teachers, however, value calm play and are major sources of children’s social and emotional growth (Jeon H. et al., 2019). In addition, the formation of children’s gender consciousness is achieved through the identification and imitation of gender roles (Erikson, 1980) that partially form the performance of male and female teachers in the workplace. As a result, both male and female ECTs are put in specific positions to meet children’s needs. Thus, the frequent physical activities carried out and specific gender roles played by male ECTs in the workplace are beneficial for releasing stress, whereas a higher level of emotional involvement and demand for female teachers may contribute to an increase of stress and burnout at work.

As for work–family conflict, according to the conservation of resources theory (COR), individuals have a limited number of personal resources (such as time, energy, and emotions) that take maximum effort to acquire, preserve, and maintain (Hobfoll et al., 2018). Compared to male teachers, female ECTs have a higher level of emotional involvement and a lower level of emotional regulation; thus, to maintain a balance between work and family, the greater emotional effort spent by female teachers may, to some extent, affect their performance when it comes to family obligations (such as taking care of their own children), leading to higher work–family conflict.

A greater level of depersonalization and fewer personal accomplishments are found in male ECTs compared to female teachers. In terms of gender differences, men are significantly more stressed regarding achievement-related challenges, whereas women are more physiologically reactive to social rejection (Stroud et al., 2002; Chen et al., 2018). This indicates that women are more stressed when there are interpersonal conflicts and challenges compared to men; to avoid social rejection, women tend to pay more attention to others at work and less to themselves, thereby buffering against the effect of depersonalization. Meanwhile, in terms of work roles, men tend to be burdened by higher social expectations; as a result, they focus more on self-achievement, especially at work. However, previous studies have underestimated the representation of male ECTs (Stroud et al., 2000; Sumsion, 2005) and their value for ECE and development, which has not been fully recognized and respected by the public. Moreover, previous studies on ECTs’ stress indicate that a wide variety of work events and the associated workload lead to negative emotional experiences (Lin et al., 2017). This may be one reason for the low personal accomplishments of male teachers.

For overall differences, compared with Ghana and Pakistan, Chinese ECTs experience the highest overall level of stress and burnout (emotional exhaustion and low personal accomplishments). Teachers in Pakistan have the lowest level of emotional exhaustion and workload, as well as the highest sense of personal accomplishments. In contrast to their Chinese and Pakistani counterparts, Ghanaian teachers experience fewer emotional demands at work and work–family conflicts. Furthermore, teachers in Ghana are less likely to undergo a depersonalization process.

First, in terms of policy requirements and constraints, China has always placed great importance on the quality of ECE and established high requirements for teachers’ quality and professional ability, as reflected in a series of typical policies. For example, the “Guidelines to Learning and Development for Children Aged 3–6″ require teachers to learn the connotations of children’s learning and development, to master the core values and key elements of each domain (health, language, society, science, and art), and to understand each child’s level of development to carry out targeted education and teaching activities (Wang, 2014) both comprehensively and systematically. The “Outline of Education Planning” requires kindergartens to strictly implement teacher qualification standards, to strengthen teacher training, and to improve teachers’ overall quality. The abovementioned policies primarily focus on ethics and professional attitudes (especially emotional attitudes and emotional involvement), children’s lives and health, care and education, educational and practical teaching ability (observation, the organization of activities and play, implementation and evaluation of the curriculum, environmental creation, etc.), reflection, and independent professional development (Pang, 2012). Although China’s policy requirements and constraints provide directions for ECTs’ professional growth, they may also be a major source of stress.

Second, in terms of cultural influences, China has been deeply influenced by Confucianism since ancient times, especially with respect to teachers. The influence of Confucian culture is reflected in the status and requirements of teachers. For example, in Confucianism, teachers and monarchs, as a country’s foundation, are in parallel with the origin of life and the nation to reflect the importance of educators. Thus, respect for teachers is an integral part of Chinese culture; as such, teachers have always shouldered important responsibilities. Accordingly, Confucianism requires teachers to be professional, knowledgeable, and outstanding while also having a pleasant personality and a strong sense of morals (Zhang, 2021). Based on the development of children in all areas, teachers need to demonstrate comprehensive teaching abilities and professional skills at work; at the same time, they need to model moral behaviors in dealing with children’s daily emotional needs; this is precisely the realistic embodiment of the requirements of Confucianism.

ECTs in China perceive more work–family conflict than family–work conflict (Yue and Ji, 2017). In traditional Chinese culture, women shoulder important family responsibilities (such as taking care of the children and housework), which forces them to invest a lot of time in family maintenance. However, nowadays, women’s values and status are recognized and respected, especially in their roles and significance at work. The integration and transmission of culture affect families’ contradictions. For ECTs, the fulfillment and balance of family duties (especially taking care of children) are particularly prominent. For example, whether teachers can balance their emotions and pay enough attention to children can be a great challenge. As such, this may be why ECTs in China have a higher risk of work–family conflict.

Although outcomes are mixed between stress and burnout, compared with Pakistan and Ghana, Chinese ECTs have the highest level of job and emotional demands, as well as a greater level of work–family conflict. Chinese teachers deal with a large amount of emotional involvement (emotional demands) at work. Due to the high level of workload and work–family conflict, when trying to balance psychological resources and work conflict, the accumulation of long-term emotional pressure may lead to emotional exhaustion. In this case, emotions may be the key factor leading to burnout. However, according to the Effort-Reward Imbalance Model (ERI), an imbalance of social exchange and reciprocity in the workplace contributes to stress and negative emotions and stress-related responses (Siegrist, 2002). Compared to Pakistan and Ghana, Chinese ECTs have the lowest level of personal accomplishments. First, China emphasizes the quality of ECE, which contributes to a high workload. However, the achievements of ECTs (such as when coping with children’s physical and mental development and changes in their behavior) are delayed and implicit (Li et al., 2019). As a result, demanding jobs bring low levels of personal fulfillment and accomplishments. Li (2018) explored the relationship between teacher-perceived social support and burnout, and found that although there are regional differences, Chinese ECTs perceive low social support with high-quality demands, and there is a negative predictive effect between social support and burnout. This indicates that a reduced sense of personal accomplishments may result from inadequate social support at the teacher level.

The situation in Pakistan is quite different. The quality of ECE in Pakistan has always been marked by deficiencies in numerous aspects, including a lack of trained teachers, resources, and awareness of the importance of ECE experiences (Zada, 2014). Although the National Education Policy (NEP) puts the quality of ECE at the core of its strategic position (Ministry of Education, 2009, p. 35), teacher education and training remain an issue. Few connections have been found between teachers’ educational institutions and district policymakers and schools. Inadequate guidance and supervision from the government and low access requirements make it difficult to improve the quality of teachers in a sustainable way. Teachers’ educational institutions are not able to provide support for teachers in connecting practice with theory (Zada, 2014). This indicates that there may be rigid teaching and negative preparation in the work of Pakistani ECTs. At the same time, due to low professional requirements for teachers at work, the workload and emotional involvement of ECTs in Pakistan are lower compared with China. Interestingly, ECTs in Pakistan reported the highest sense of personal accomplishments compared to ECTs in China and Ghana. Culture may serve as an important factor in the conflict between low job requirements and high personal accomplishments. Pakistan is a hierarchical country with a huge gap between the rich and poor (Zhang, 2011). As the state religion, Islam advocates for peace and equality, which builds solid confidence among ordinary people (Du, 2012). Compared with the strict requirements and constraints of Confucianism for Chinese teachers, Islamic culture endows Pakistani teachers with a positive attitude when coping with potential work stress, which may contribute to a higher sense of personal accomplishments. However, Pakistan remains a traditional patriarchal society, and ordinary people are far more loyal to their families and tribes than to the state (Du, 2012). As a typical collectivist country, compared with China, teachers in Pakistan might not pay much attention to individual achievements at work while attaching importance to family interests. However, even if loyalty to one’s family can partially explain the reasons for work–family conflict among ECTs in Pakistan, as a female-dominated profession, the role of gender differences is worth exploring. According to Zhang (2011), women in Pakistan rarely work outside the home; instead, their main responsibility is raising children and managing household affairs. Therefore, compared with men, Pakistani women are more likely to experience work–family conflict, which is also typical for ECTs. As for the high depersonalization among Pakistani ECTs compared to Chinese and Ghanaian ECTs, the lower quality of ECE and a lack of professional development opportunities may be the main reason. A series of factors affects the quality of ECE, such as the teacher-child relationship (Koles et al., 2013; Ansari et al., 2022), teaching efficacy (Li et al., 2019), teachers’ social emotional capacity (Buettner et al., 2016), class, and organizational climate (Li et al., 2019; Ansari et al., 2022); these factors are closely related to burnout and well-being among ECTs. Insufficient ECE quality results from limited resources and professional development opportunities, which may lead to negative professional attitudes toward work and reduced self-engagement. Furthermore, teacher qualifications are important for teaching practices, children’s learning, and teachers’ well-being (Croninger et al., 2007; Early et al., 2007; Jeon H. et al., 2019). Conflict-related emotional responses to stress (lower emotional exhaustion, higher personal accomplishments but inadequate professionalism, and negative professional attitudes) may contribute to an accumulated sense of self-dissociation and ultimately lead to high depersonalization.

Similar to Pakistan, the quality of ECE and teacher development are also troubling in Ghana. In terms of policy, Ghana emphasizes basic education. For instance, in order to ensure that children from low socioeconomic families benefit from basic education, kindergarten was incorporated into the formal FCUBE system (Agbenyega, 2018), while the Children’s Act (1998) and the Early Childhood Care and Development (ECCD) policy UNICEF (2004) express the concern and attention paid to the quality of ECE. However, teachers in most early childhood learning programs in Ghana use rote-learning methods, including academic tests to measure school readiness (Asare and Nti, 2014). Children’s passive learning is evident in rigorous daily routines, although ECE policies and practices advocate for play-based constructivist teaching (Daniels, 2001; Kotar, 2014). Furthermore, curricula, teaching, and assessment for children are rigid, leaving no room for adjustment to meet individual children’s developmental needs (Agbenyega and Deku, 2011; Agbenyega, 2014; Asare and Nti, 2014). In terms of coping strategies for children’s behaviors, corporal punishment remains common (Agbenyega, 2018). Clear evidence has proven the huge gap between policy direction and practice, with teachers being the key influencing factor. ECTs in Ghana fail to provide appropriate emotional support, and rigid teaching methods hamper positive emotional communication. This is not only greatly affects teachers’ emotional involvement, but also promotes the generation of negative emotional responses. There may also be a reason why Ghanaian teachers are more emotionally exhausted, despite having a lower level of emotional involvement (emotional demands) than their Pakistani counterparts. Religious belief may also have a potential moderating effect on the emotional exhaustion of ECTs in Pakistan.

Moreover, ECTs in Ghana had the lowest level of work–family conflict compared with their Chinese and Pakistani peers. In terms of social values, a collectivist society with cohesion and loyalty to one’s family contributes to mutual support within the family (Hofstede, 2011). Furthermore, Ghanaians may have the lowest level of work–family conflict due to absence of gender restrictions compared to Pakistan and a lower level of workload compared to China. Compared to China and Pakistan, the depersonalization of ECTs in Ghana is not apparent. Collectivism in Ghana is characterized by a social ontogenetic paradigm that emphasizes interdependent relationships, so teachers may prefer to internalize burnout (through emotional exhaustion) instead of affecting relationships in the workplace (through depersonalization) (Lee and Wolf, 2019). This not only explains the lower level of depersonalization among Ghanaian ECTs, but also illuminates a new direction for coping with a higher level of emotional exhaustion. Compared with social values, Christian religious beliefs may strengthen a teacher’s attention to themselves as an individual and may influence the frequency of depersonalization at work.

Ghana and Pakistan share a number of common problems, including disconnection between stakeholders in the educational system that results in a lack of resources and an insufficient awareness of emotional involvement among educators. This disconnection may contribute to failure in receiving positive or appropriate feedback from organizations or the children they teach, and might even be replaced by negative and delayed feedback. This will not only threaten a teacher’s emotional well-being and weaken their motivation to teach (Geake, 2009), but also may lead to burnout thus hindering professional development. For instance, direct and negative feedback is positively correlated with burnout (Kremanshahi and Pishghadam, 2022). Therefore, the low level of emotional involvement of ECTs in Ghana and Pakistan may be related to their lack of motivation. More importantly, the continuation of this disconnection might have a long-term impact on teachers’ burnout and well-being. As close connections among stakeholders in the educational system are established, and teachers are able to acquire resources and coping strategies for children’s behaviors in practice, teachers might not only improve their motivation in teaching but also reduce their level of burnout, therefore leading to ongoing professional development. Some scholars have explored the role of time perspectives in teacher burnout; the negative outlook on the past and present fatalistic time perspectives are positively correlated with burnout, while the positive and optimistic perspectives will in turn significantly reduce the level of teacher burnout (Naji Meidani et al., 2019). The life-span development theory indicates that individual growth occurs in the interaction of selection, optimization, and compensation with a change in age, and the dynamic state of resources serves the plasticity of development (Baltes, 1997). Consequently, either positive responses or resources and support tailored to the individual teacher may have a positive impact on their burnout and well-being.

In addition, teachers in Ghana and Pakistan may suffer from inadequate motivation resulting from gaps in resources. Stroking may be one solution in teacher training. Stroking in teaching refers to acknowledging teachers’ presence and care for children (Pishghadam and Farkhondehfal, 2017; Pishghadam and Karami, 2017), which may not only promote the positive feedback that teachers acquire, but also encourage the teaching-student relationship (Pishghadam and Khajavy, 2014; Pishghadam et al., 2019). This kind of support meets the needs of emotionality as a profession and contributes to ongoing professional growth with motivation.

Finally, in line with previous studies, the relationship between stress and burnout is complex. In general, the work of ECTs in China is highly demanding and requires a lot of emotional involvement, which may result in emotional exhaustion. At the same time, work–family conflict may aggravate the emotional exhaustion of Chinese teachers, although for various reasons, Pakistani ECTs face the same risk as their Chinese peers. Because of the relationship between work and family, ECTs in China and Pakistan are also experiencing a loss of sense of self or environment (depersonalization). We assumed that ECTs in China and Ghana may suffer from an imbalance between work efforts and rewards as participants in both countries had a lower sense of personal accomplishments.

## Limitations

Although this study analyzes ECTs’ characteristics of stress and burnout in developing countries from a cross-cultural perspective, there are still some directions for future research. First, the existing measurement instruments for gaging ECTs’ stress and burnout were developed in Europe and the US (Erdiller and Doğan, 2015; Jeon H. et al., 2019; Schaack et al., 2020). Given cultural influences, indicators are expected to be further enriched and improved in cross-regional and cross-cultural contexts. Second, each portion of the sample was recruited from a certain region in each country, which may affect the representativeness to some extent. Therefore, future studies should increase the representativeness of their samples while considering correlations. For instance, participants from China mostly came from the east, which advocates for Confucian culture, but followers of Islam are found in China’s west. Similarly, although Islam is Pakistan’s national religion, neighboring India, where most of the population is Hindu, may have some influence on Pakistan’s ECTs. In nearby regions, similarities and differences of faith in Ghana, Nigeria, and Ivory Coast are worth exploring. Third, this study performed a comprehensive comparative analysis of ECTs’ stress and burnout in China, Ghana, and Pakistan by starting with the educational system, beliefs, and values. However, from the perspective of cultural analysis, the current framework is weak. Some scholars have examined teachers’ burnout using the cultural dimensions framework, and cultural dimensions such as uncertainty avoidance, indulgence/restraint, and masculinity/femininity have a certain predictive effect on burnout (emotional exhaustion, depersonalization, and personal achievements) (Sabouri and Pishghadam, 2016). Cultural analysis, as a social construct, has room for expansion, and the comparison of individualism/collectivism and beliefs might not be enough to fully respond to the problem. For example, this study used biological gender analysis to reflect ECTs’ stress and burnout. In specific environmental and cultural contexts, the analytical perspectives on masculinity/femininity may produce different outcomes. Fourth, this study carried out an overall analysis of the main stressors and burnout among ECTs in the three countries from a somewhat macro perspective. If the focus is on emotionality as a profession, the problem will become more complicated. For example, personality traits (Grandey and Gabriel, 2015) and emotional intelligence (Yin et al., 2019) will affect teachers’ emotional regulation process and contribute to a change in stress levels. At the same time, personality type and emotional intelligence have a predictive effect on teachers’ burnout (Pishghadam and Sahebjam, 2012). Due to the differences in individual growth experiences and cultural backgrounds, these factors may have a stronger predictive effect on ECTs’ stress and burnout. Fifth, to improve ECTs’ psychological health and well-being, the role of positive psychology in stress and burnout is worth investigating further. For example, flourishing (including emotional and psychological well-being) mediates the relationship between teachers’ psychological capital and burnout (Diener, 2000; Freire et al., 2020). Therefore, flourishing may play a significant role in reducing teachers’ burnout. A study of Japanese workers showed that physiological workload was positively correlated with flourishing, while psychological workload was negatively correlated (Hori et al., 2019). Although there are occupational differences, the partial association between flourishing, stress, and burnout provides a new dynamic perspective for the improvement of ECTs’ well-being. An individual’s perceived level of stress may have positive and negative effects that can be transformed into each other instead of a single contradiction. Under moderate stress, individuals may be able to maintain a high level of well-being by managing stressors and emotions. Additionally, with the possible dynamic transformation of stress and burnout, another construct worth scrutinizing in positive psychology is stroking. Care is one of the professional attributes in ECTs with the requirement of ECE quality. Stroking concentrates on care in an interactive relationship (Pishghadam and Farkhondehfal, 2017; Pishghadam and Karami, 2017), and motivates individuals to receive strokes (Pishghadam and Khajavy, 2014; Pishghadam et al., 2019). Teachers and children may benefit from further research on the role and specific effects of stroking on teaching and learning.

## Conclusion

From a cross-cultural angle, this study comparatively analyzed the characteristics of stress and burnout among ECTs in different cultural settings and educational systems in three developing countries (China, Ghana, and Pakistan). Compared with prior studies on cross-cultural teachers’ stress, which mostly focused on comparisons among developed countries (Klassen et al., 2010; Hong and Zhang, 2019), this study fills a gap in the literature on comparative studies regarding stress and burnout in developing countries. This study revealed workplace characteristics and circumstances for ECTs by comparing similarities and differences, and by performing factor analysis on stress and burnout among ECTs in developing nations. As a result, policymakers and stakeholders in different countries may be encouraged to improve ECE quality and the well-being of ECTs. In addition, past studies on ECTs’ stress have rarely focused on gender differences. This study takes gender as the main influencing factor and explores its effect on ECTs’ stress and burnout. This study highlights and verifies “emotionality” in ECTs’ profession, but also suggests ways in which policymakers and practitioners can support ECTs’ emotional health and daily work.

## Data availability statement

The original contributions presented in the study are included in the article/supplementary material, further inquiries can be directed to the corresponding author.

## Ethics statement

The studies involving human participants were reviewed and approved by Ethics Review Form for Studies at the Zhejiang Normal University (ZJNU). The patients/participants provided their written informed consent to participate in this study.

## Author contributions

JQ developed the study concept. SC implemented the study, collected and analyzed the data, and drafted the manuscript. SN collected and analyzed the data. SC and JQ revised the manuscript. All authors interpreted the results and approved its final version for submission.

## Funding

This study was supported by the Key Project of National Social Science Foundation of China (18ASH015).

## Conflict of interest

The authors declare that the research was conducted in the absence of any commercial or financial relationships that could be construed as a potential conflict of interest.

## Publisher’s note

All claims expressed in this article are solely those of the authors and do not necessarily represent those of their affiliated organizations, or those of the publisher, the editors and the reviewers. Any product that may be evaluated in this article, or claim that may be made by its manufacturer, is not guaranteed or endorsed by the publisher.
